# Mild (not severe) disc degeneration is implicated in the progression of bilateral L5 spondylolysis to spondylolisthesis

**DOI:** 10.1186/s12891-018-2011-0

**Published:** 2018-04-02

**Authors:** Vivek A. S. Ramakrishna, Uphar Chamoli, Luke L. Viglione, Naomi Tsafnat, Ashish D. Diwan

**Affiliations:** 1 Spine Service, Department of Orthopaedic Surgery, St. George & Sutherland Clinical School, University of New South Wales Australia, Kogarah, Sydney, NSW 2217 Australia; 2 School of Biomedical Engineering, University of Technology Sydney, Ultimo, NSW 2007 Australia; 3 School of Mechanical and Manufacturing Engineering, University of New South Wales Australia, Kensington campus, Sydney, NSW 2052 Australia

**Keywords:** Spondylolysis, Spondylolisthesis, Biomechanical instability, Disc degeneration, Intervertebral disc

## Abstract

**Background:**

Spondylolytic (or lytic) spondylolisthesis is often associated with disc degeneration at the index-level; however, it is not clear if disc degeneration is the cause or the consequence of lytic spondylolisthesis. The main objective of this computed tomography based finite element modelling study was to examine the role of different grades of disc degeneration in the progression of a bilateral L5-lytic defect to spondylolisthesis.

**Methods:**

High-resolution computed tomography data of the lumbosacral spine from an anonymised healthy male subject (26 years old) were segmented to build a 3D-computational model of an INTACT L1-S1 spine. The INTACT model was manipulated to generate four more models representing a bilateral L5-lytic defect and the following states of the L5-S1 disc: nil degeneration (NOR LYTIC), mild degeneration (M-DEG LYTIC), mild degeneration with 50% disc height collapse (M-DEG-COL LYTIC), and severe degeneration with 50% disc height collapse(S-COL LYTIC). The models were imported into a finite element modelling software for pre-processing, running nonlinear-static solves, and post-processing of the results.

**Results:**

Compared with the baseline INTACT model, M-DEG LYTIC model experienced the greatest increase in kinematics (Fx range of motion: 73% ↑, Fx intervertebral translation: 53%↑), shear stresses in the annulus (Fx anteroposterior: 163%↑, Fx posteroanterior: 31%↑), and strain in the iliolumbar ligament (Fx: 90%↑). The S-COL LYTIC model experienced a decrease in mobility (Fx range of motion: 48%↓, Fx intervertebral translation: 69%↓) and an increase in normal stresses in the annulus (Fx Tensile: 170%↑; Fx Compressive: 397%↑). No significant difference in results was noted between M-DEG-COL LYTIC and S-COL LYTIC models.

**Conclusions:**

In the presence of a bilateral L5 spondylolytic defect, a mildly degenerate index-level disc experienced greater intervertebral motions and shear stresses compared with a severely degenerate index-level disc in flexion and extension bending motions. Disc height collapse, with or without degenerative changes in the stiffness properties of the disc, is one of the plausible re-stabilisation mechanisms available to the L5-S1 motion segment to mitigate increased intervertebral motions and shear stresses due to a bilateral L5 lytic defect.

**Electronic supplementary material:**

The online version of this article (10.1186/s12891-018-2011-0) contains supplementary material, which is available to authorized users.

## Background

Isthmic spondylolysis (or lytic defect) is characterised by a bony defect of the pars interarticularis and occurs most commonly as a bilateral defect in the L5 vertebra [1, 2]. The mechanism for the progression of the lytic defect to spondylolisthesis remains unclear despite viable qualitative theories supported with clinical radiographic evidence [3–9].

Abnormal spinopelvic morphology and orientation measured through parameters such as pelvic incidence, sacral slope, pelvic tilt may create a biomechanical environment leading to the development of a pars defect and its progression to spondylolisthesis [3–6, 9]. In children and adolescents, the progression of the defect to spondylolisthesis is attributed to growth plate injury which may perpetuate to epiphyseal ring separation [8, 10]. Slippage in skeletally immature spines is not attributed to any disc degeneration, but rather growth plate lesions [7].

Lytic spondylolisthesis is often associated with disc degeneration at the olisthetic segment; however, it is not clear whether disc degeneration is the cause or consequence of lytic spondylolisthesis [11]. Clinical evidence suggests that disc degeneration occurs more rapidly and prematurely in the presence of lytic spondylolisthesis [11, 12]. Szypryt et al. (1988) compared disc degeneration at the olisthetic segment in 40 consecutive patients with an age-and-sex matched control group of 40 asymptomatic volunteers. The authors observed that over the age of 25 years, the prevalence of disc degeneration was significantly higher in the spondylolytic group compared with the control group [11]. Seitsalo et al. (1997) in their long-term follow-up study of operatively and conservatively treated isthmic spondylolisthesis patients reported a strong correlation between the severity of disc degeneration and the degree of slippage in the conservatively treated cohort. Progression to spondylolisthesis in adults is almost always attributed to disc degeneration [11, 13]. Case studies, however, have cited the rare progression of pars defect to vertebral slippage in adults without any associated disc degeneration, adding to the ambiguity around the role of disc degeneration in the pathomechanism for slippage progression [14, 15].

The intervertebral disc plays an important role in resisting shear and torsional forces acting on the spinal column during physiological bending motions. Biomechanical studies on cadaveric lumbar spines with healthy intervertebral discs have shown increased kinematics at the index-level following a bilateral lytic defect [16–18]. The loss of posterior tension band in the lumbar spine following the defect may redirect excessive shear and torsional forces on to the index-level disc, which may predispose the disc to accelerated or premature degeneration [19]. With progressive disc degeneration, the shear and torsional stiffness of the disc may get compromised, which in addition to the loss of posterior tension band, may perpetuate to spondylolisthesis. However, from a biomechanical standpoint, it remains unclear how different grades of disc degeneration may be implicated in the progression of a lytic defect to spondylolisthesis. Yong-Hing and Kirkaldy-Willis (1983) suggested that a degenerative progression occurs in the intervertebral disc from a state of hypermobility in the early stages of degeneration to hypomobility in the later stages [20]. Disc degeneration grades III and IV (Thomson’s grading) characterised by nuclear clefts, radial and concentric tears in the annulus fibrosus have been reported to cause kinematic instability in lumbar motion segments, whereas grade V degeneration characterised by disc height collapse and osteophyte formation resulted in biomechanical stability in the segments [21].

Here we use computed tomography (CT) data from a healthy human subject and nonlinear finite element modelling (FE) to assemble three-dimensional models of the lumbosacral spine to investigate the role of different grades of disc degeneration in the progression of a bilateral L5 lytic defect to spondylolisthesis. We hypothesised that a mildly degenerated disc is not able to arrest hypermobility following a bilateral lytic defect in the L5 vertebra and therefore the defect is disposed to progress to spondylolisthesis; however, in a severely degenerated disc, hypomobility is sufficient to stabilise the motion segment and limit the progression of the defect to spondylolisthesis.

## Methods

### Image segmentation and 3D model generation from CT data

**Fig. 1:**
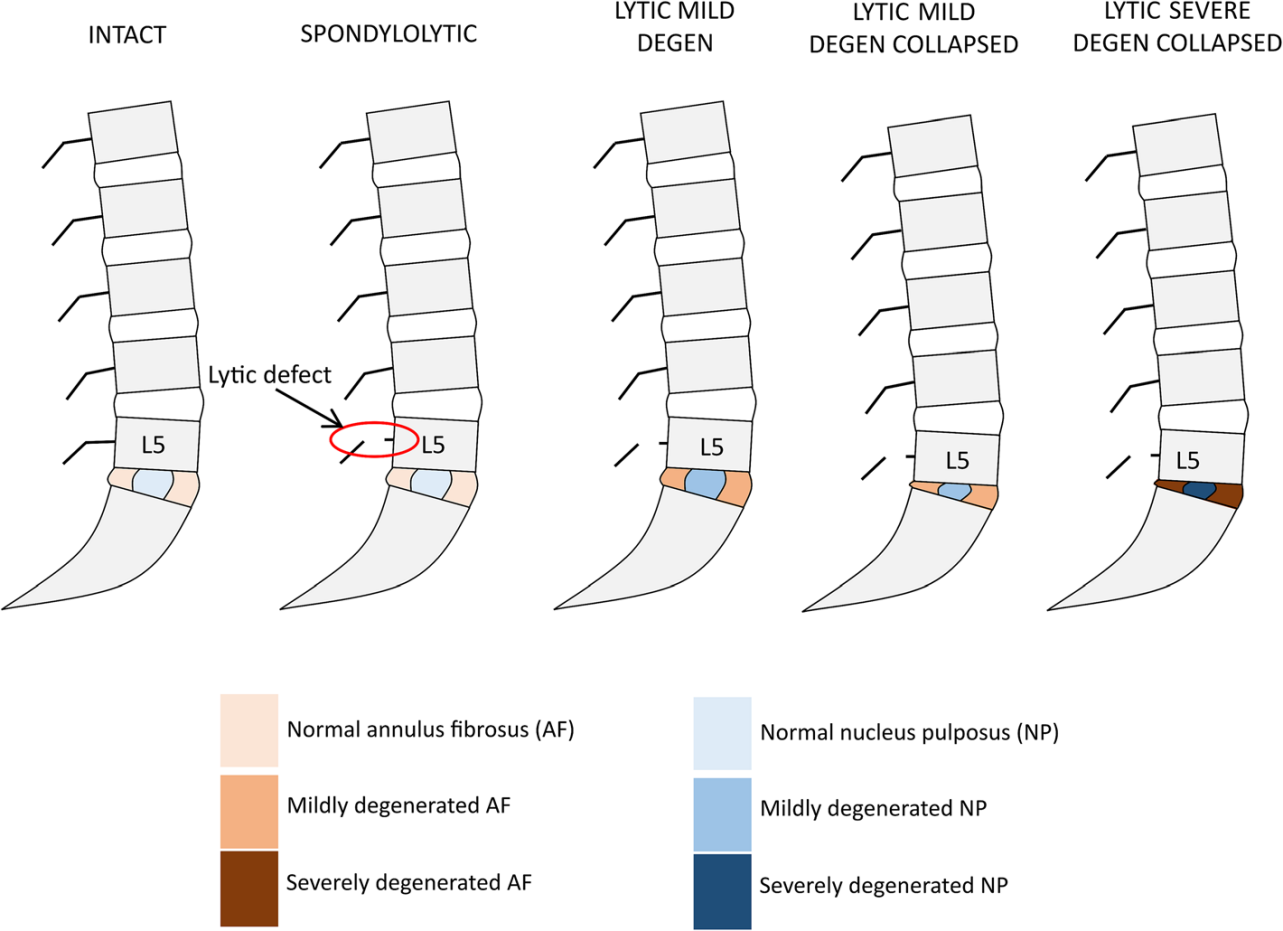
Pictorial representation of the five finite element models of lumbosacral spine representing various states of the L5-S1 intervertebral disc with or without a bilateral spondylolytic defect at L5. Compared with the INTACT model, a disc height loss of 50% in the mid-sagittal plane was modelled in the Lytic-Mild-Degen-Collapsed and Lytic-Severe-Degen-Collapsed models

Prior approval from UNSW Human Research Ethics Advisory Panel was obtained for the use of retrospectively acquired CT data from a human subject (UNSW-NRR-HC16754). High-resolution lumbosacral spine CT data (437 axial cuts, 512 × 512 pixel resolution, voxel dimensions: 0.30 × 0.30 × 0.50 mm) from an anonymised healthy male subject (26 years old) were obtained in DICOM (Digital Imaging and Communications in Medicine) file format from Carl Bryan Radiology (St. George Private Hospital, Sydney, Australia). Using previously published protocols, the CT data were imported into image processing software Avizo Standard (vers. 8.1, FEI Visualization Sciences Group, Hillsboro, USA) for the segmentation of anatomical regions of interest, and subsequent generation and refinement of surface and volumetric meshes [22]. The segmented CT data were manipulated to generate a bilateral L5 spondylolytic defect model by deleting pixels from the L5 pars region (NOR LYTIC), creating an approximately 2-mm (mm) wide fracture gap between the bony fragments. Three more L5 spondylolysis models were built representing: mild disc degeneration (M-DEG LYTIC), mild disc degeneration with a disc height collapse (M-DEG-COL LYTIC), and severe disc degeneration (S-COL LYTIC) as illustrated in Fig. 1.

The intervertebral disc is a composite structure comprising the annulus fibrosus and the nucleus pulposus, which degenerates accordingly with morphological and material property changes to both. In accordance with published material property data for normal and degenerated discs, the shear modulus for the annulus fibrosus (AF) was increased (mild: + 25%, severe: + 50%) and the Poisson’s ratio for the nucleus pulposus (NP) was decreased (mild: − 13%, severe: − 34%) compared with the INTACT state in the FE models representing L5-S1 disc degeneration [23–25]. Disc height collapse was modelled as a 50% reduction in disc height measured in the mid-sagittal plane. The segmented CT data were manipulated to represent disc height collapse by moving the sacrum superiorly to reduce sagittal mid-disc height at the L5-S1 level from 14.35 mm to 6.64 mm while preserving the L5-S1 facet articulation.

### Modelling annulus Fibres and ligaments

Volumetric meshes in Nastran file format (.nas) representing different variants of the lumbosacral spine model were imported into the FE modelling software Strand7 (vers. 2.4.6, Strand7 Pty. Ltd., Sydney, Australia). The annulus fibrosus was modelled per previously published protocols as a composite structure comprising concentric layers (*n* = 4) of criss-cross collagen fibres embedded within a homogenous ground substance, with the ends of the fibres rigidly anchored in the superior and inferior endplates [22]. Ligaments were modelled using cylindrical beam elements, with the attachment and insertion sites based on previously recorded anatomical observations and published protocols [22, 26]. The type and number of elements used in assembling the five FE models are presented in Table 1.

**Table 1:** Type and corresponding number of elements used in different finite element models of the lumbosacral spine

		INTACT	NOR LYTIC	M-DEG LYTIC	M-DEG-COL LYTIC	S-COL LYTIC
Nodes		308,539	320,256	320,256	382,025	382,025
Brick element (4-noded tetrahedrons)		1,454,554	1,522,196	1,522,196	1,811,798	1,811,798
Facet articulation (Nonlinear point contact elements)		5 per joint	5 per joint	5 per joint	5 per joint	5 per joint
Bilateral L5 lytic defect (Zero gap elements)		0	25 per side	25 per side	25 per side	25 per side
Annulus fibres (cylindrical beam elements)	L1-L2	(O) 312–321–293-266 (I)	(O) 289–272–229-224 (I)	(O) 289–272–229-224 (I)	(O) 350–311–279-262 (I)	(O) 350–311–279-262 (I)
	L2-L3	(O) 362–385–347-287 (I)	(O) 271–266–254-168 (I)	(O) 271–266–254-168 (I)	(O) 359–316–268-255 (I)	(O) 359–316–268-255 (I)
	L3-L4	(O) 330–298–232-240 (I)	(O) 265–244–212-189 (I)	(O) 265–244–212-189 (I)	(O) 395–318–269-258 (I)	(O) 395–318–269-258 (I)
	L4-L5	(O) 308–331–303-237 (I)	(O) 251–218–233-163 (I)	(O) 251–218–233-163 (I)	(O) 385–322–284-253 (I)	(O) 385–322–284-253 (I)
	L5-S1	(O) 243–258–236-219 (I)	(O) 202–192–169-167 (I)	(O) 202–192–169-167 (I)	(O) 436–352–341-291 (I)	(O) 436–352–341-291 (I)
Ligaments per level (cylindrical beam elements)	ALL	14	14	14	14	14
	PLL	6	6	6	6	6
	TL	16	16	16	16	16
	LF	18	18	18	18	18
	ISL	9	9	9	9	9
	SSL	4	4	4	4	4
	CL	24 per joint	24 per joint	24 per joint	24 per joint	24 per joint
	ILL	20	20	20	20	20
	LSL	22	22	22	22	22

The concentric rings of annulus fibres were modelled using nonlinear beam elements

(O) Layer1 (outermost)-Layer2-Layer3-Layer4 (innermost) (I); *ALL* Anterior Longitudinal Ligament, *PLL* Posterior Longitudinal Ligament, *TL* Transverse Ligament, *LF* Ligamentum Flavum, *ISL* Interspinous Ligament, *SSL* Supraspinous Ligament, *CL* Capsular Ligament, *ILL* Iliolumbar Ligament, *LSL* Lumbosacral Ligament

### Facet joint articulations and pars Interarticularis fracture gap

The compressive load bearing characteristics of the bony articulating pillars at each facet joint was modelled using nonlinear *Point Contact-Tension* elements in Strand7 (*n* = 5 per joint), which were normally oriented and uniformly distributed over the bony articulating surfaces. The bilateral pars interarticularis fracture gap in the NOR LYTIC, M-DEG LYTIC, M-DEG-COL LYTIC, and S-COL LYTIC models was connected using nonlinear *Point Contact-Zero Gap* elements (*n* = 25 each side, compressive stiffness only) to allow for load transfer between the fractured fragments in the event of gap closure during simulated bending motions.

### Loads and boundary constraints

In all the models, a centre node on the anterior surface of the sacral mass was fixed in all translational and rotational degrees of freedom (DOFs). Bending motions were simulated using a cross-beam construct accommodated on the L1 superior endplate by means of a surface cap, both of which were assigned material properties of stainless steel (E = 200 GPa, ν =0.25). A force couple was applied to the anterior and posterior ends of the cross-beam to simulate flexion (Fx) and extension (Ex) bending. The models were loaded in pure unconstrained moments (without any compressive preload) with step-wise increments in load from 1.0 N-metre (Nm) to 10 Nm. The pre-processed FE models were solved for geometry, material, and boundary nonlinearities using the nonlinear static solver in Strand7.

### Material property values

Previously published material property values calibrated against in vitro biomechanical testing data were assigned to brick elements representing the intervertebral disc, beam elements representing the primary ligaments, and non-linear point contact elements representing facet articulation between the bony pillars (see Additional file 1). [22]

## Results

All the results were analysed at peak Fx and Ex bending loads (10 Nm) in the five FE models.

### L5-S1 range of motion (RoM)

**Fig. 2:**
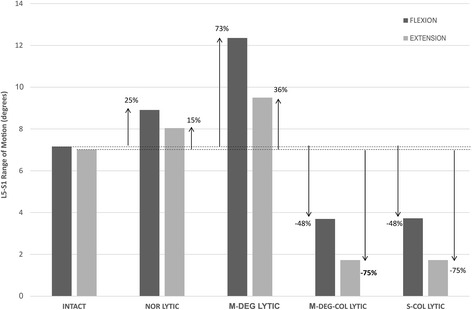
L5-S1 range of motion (RoM) in flexion (Fx) and extension (Ex) bending at peak load of 10 Nm. Compared with the INTACT model, the greatest increase in Fx and Ex RoMs was observed in the mildly-degenerate lytic defect model (M-DEG-LYTIC)

The L5-S1 Fx and Ex RoM results are presented in Fig. 2. From the baseline INTACT state, the greatest increase in Fx and Ex RoMs was observed in the M-DEG LYTIC model (Fx: 7.2° (INTACT) to 12.4° (M-DEG LYTIC); Ex: 7.0° (INTACT) to 9.5° (M-DEG LYTIC)).

### L5-S1 Interpedicular kinematics

The L5-S1 interpedicular kinematics in Fx and Ex were evaluated per published protocols [16, 27]. The in-plane component of the interpedicular travel (IPT) vector was projected onto the L5-S1 mid-discal plane to evaluate the relative posteroanterior translatory motions between the pedicles on the adjacent vertebrae.

**Fig. 3:**
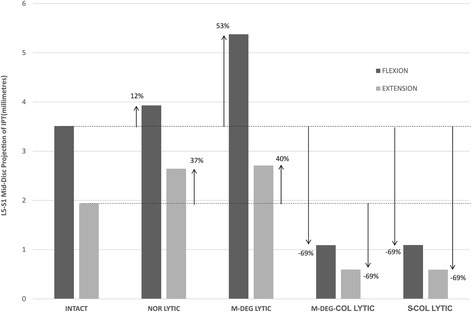
Mid-discal plane projections of the L5-S1 interpedicular travel (IPT) vector in flexion (Fx) and extension (Ex) bending at peak load of 10 Nm. Compared with the INTACT state, the greatest increase in mid-discal plane IPT projections in Fx and Ex was observed in the mildly-degenerate lytic defect model (M-DEG-LYTIC)

The L5-S1 mid-discal plane projections of the IPT vector in Fx and Ex in the five models are presented in Fig. 3. From the baseline INTACT state, the greatest increase in IPT projections on to the mid-disc plane in Fx and Ex were observed in the M-DEG LYTIC model (Fx: 3.5 mm (INTACT) to 5.4 mm (M-DEG LYTIC); Ex: 1.9 mm (INTACT) to 2.7 mm (M-DEG LYTIC)).

### Normal stresses in the L5-S1 AF

**Fig. 4:**
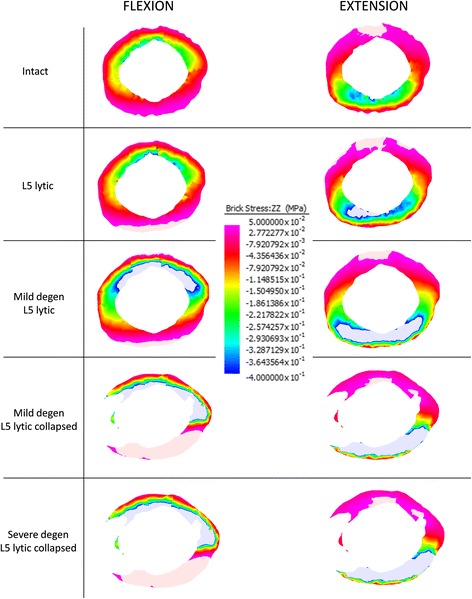
Colour coded compressive (−) and tensile (+) stress distribution in the annulus fibrosus of the L5-S1 disc in the mid discal plane at peak flexion (Fx) and extension (Ex) bending loads of 10 Nm. In both Fx and Ex bending, models with a 50% loss in disc height (Mild degen L5 lytic collapsed and Severe degen L5 lytic collapsed) recorded the greatest increase in compressive and tensile stresses from the baseline Intact state

**Fig. 5:**
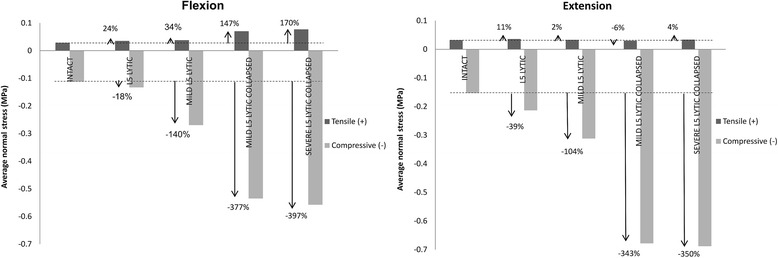
Average values of compressive (−) and tensile (−) stresses in the annulus fibrosus of the L5-S1 disc at peak flexion (Fx) and extension (Ex) loads of 10 Nm. Regardless of the bending load and model type, the annulus fibrosus experienced a significantly higher average compressive stress compared with the average tensile stress

Colour coded compressive (−) and tensile (+) stress distribution in the L5-S1 AF in the mid-axial plane of the L5-S1 disc during Fx and Ex is presented in Fig. 4. The average values for the compressive and tensile stresses in the L5-S1 AF during Fx and Ex is presented in Fig. 5.

The greatest increase in the average normal stress from the baseline INTACT state was observed in M-DEG-COL LYTIC and S-COL LYTIC models. During Fx, average compressive stress increased from 0.11 (± 0.20) MPa in the INTACT state to 0.54 (± 1.22) MPa in the M-DEG-COL LYTIC model and 0.56 (± 1.23) MPa in the S-COL LYTIC model. During Ex, average compressive stress increased from 0.15 (± 0.21) MPa in the INTACT state to 0.68 (± 1.60) MPa in the M-DEG-COL LYTIC model and 0.69 (± 1.57) MPa in the S-COL LYTIC model.

### Shear stresses in the L5-S1 AF

**Fig. 6:**
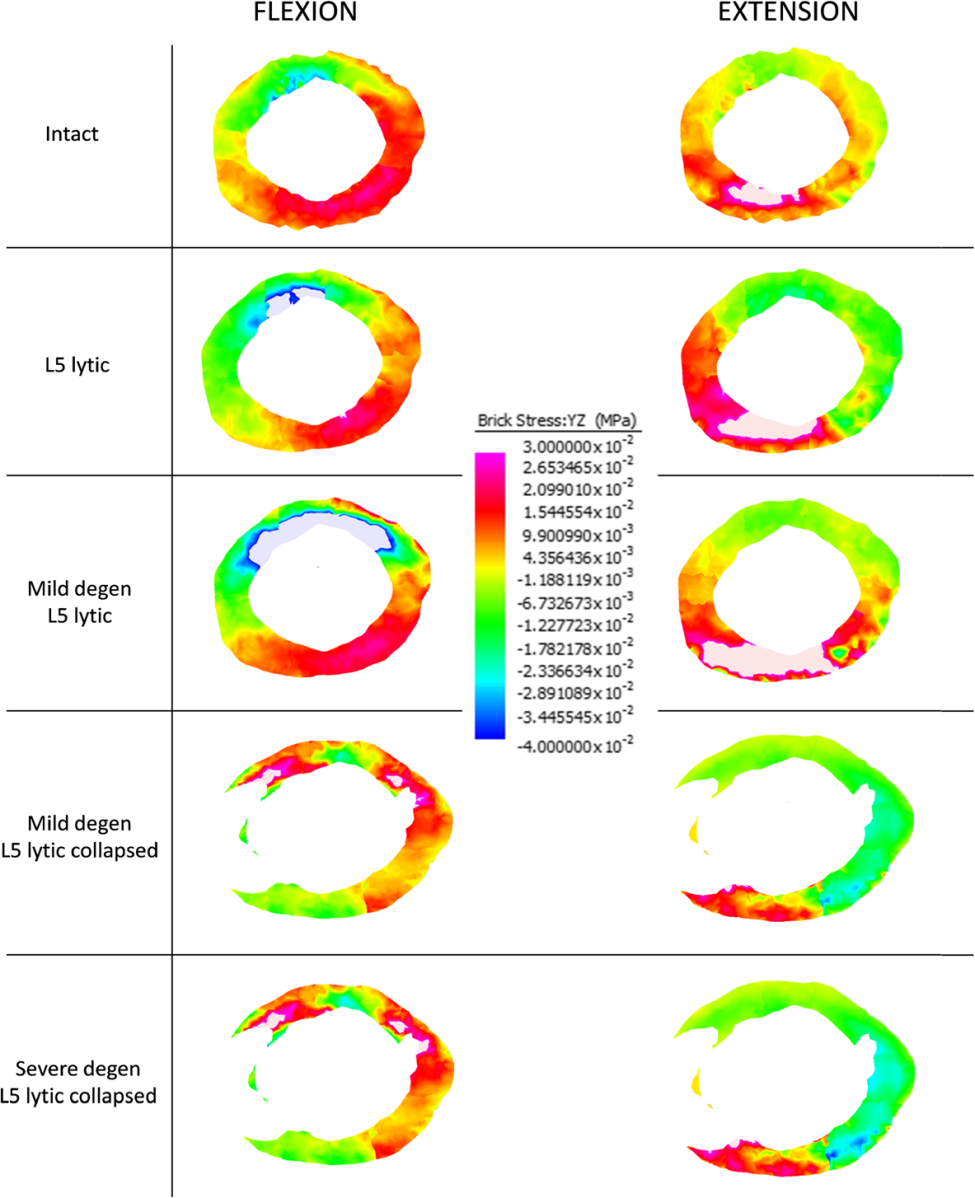
Colour coded posteroanterior (+) and anteroposterior (−) shear stress distribution in the annulus fibrosus of the L5-S1 disc in the mid discal plane at peak flexion (Fx) and extension (Ex) bending loads of 10 Nm. In both Fx and Ex bending, the mildly degenerate L5-lytic model without any disc height loss recorded the greatest increase in shear stresses from the baseline Intact state

**Fig. 7:**
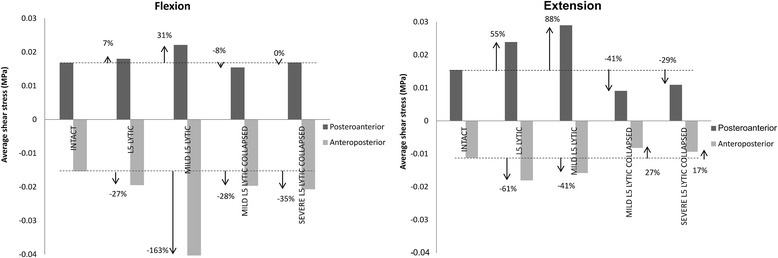
Average values of posteroanterior (+) and anteroposterior (−) shear stresses in the annulus fibrosus of the L5-S1 disc at peak flexion (Fx) and extension (Ex) loads of 10 Nm. The mildly degenerate L5-lytic model experienced the greatest increase in average anteroposterior stress in Fx and average posteroanterior stress in Ex from the baseline INTACT state

Colour coded shear stress distribution in the L5-S1 AF in the mid-axial plane of the L5-S1 disc during Fx and Ex is presented in Fig. 6. The average values for the posteroanterior and anteroposterior shear stress in the L5-S1 AF during Fx and Ex is presented in Fig. 7.

The greatest increase in shear stresses from the baseline INTACT state was observed in M-DEG LYTIC models. During Fx, average anteroposterior shear stress increased from 0.015 (± 0.016) MPa in the INTACT state to 0.04 (± 0.046) MPa in the M-DEG LYTIC model. During Ex, average posteroanterior stress increased from 0.015 (± 0.014) MPa in the INTACT state to 0.029 (± 0.029) MPa in the M-DEG LYTIC model.

### Axial strain in iliolumbar ligament (ILL) fibres

Table 2 Shows mean axial strain in the ILL fibres during peak Fx load. The ILL fibres did not experience any positive strain during peak ex load.

**Table 2:** Mean axial strain in the iliolumbar ligament (ILL) fibres in different models during peak flexion load of 10 Nm

	Mean axial strain ILL fibres (± standard deviation)
Intact	5.91 (±1.87) %
Normal L5 Lytic	8.01 (±1.89)%
Mildly Degenerate L5 Lytic	11.25 (±1.91)%
Mildly Degenerate L5 Collapsed Lytic	1.91 (±0.91)%
Severely Degenerate L5 Collapsed Lytic	1.90 (±0.90)%

The greatest increase in mean axial strain from the baseline Intact state was observed in the mildly degenerate L5 lytic model

## Discussions

The main objective of this biomechanical study was to examine the role of disc degeneration in the progression of a bilateral L5 spondylolytic defect to spondylolisthesis. The natural course of isthmic spondylolisthesis has been associated with disc degeneration and spontaneous stabilisation of the olisthetic segment, but the question whether disc degeneration is the cause or the consequence of vertebral slippage remains open [12].

The results from the present study confirm our hypothesis that in the presence of a bilateral L5 lytic defect, motion segments with a mildly degenerate index-level disc are at a greater risk for vertebral slippage compared to motion segments with a severely degenerate index-level disc. Compared with the baseline INTACT model, M-DEG LYTIC model experienced the greatest increase in kinematics (Fx ROM: 73% ↑; Fx IPT mid-discal projection: 53%↑), shear stresses in the annulus (Fx anteroposterior: 163%↑; Fx posteroanterior: 31%↑), and average strain in the ILL fibres (Fx: 90%↑). The S-COL LYTIC model experienced a decrease in mobility (Fx ROM: 48% ↓; Fx IPT mid-discal projection: 69%↓) and an increase in normal stresses in the annulus (Fx Tensile: 170%↑; Fx Compressive: 397%↑) compared with the INTACT model. The results are corroborated by previous clinical findings for conservatively treated L5-S1 lytic defect patients which revealed immobility of the motion segment with severe degeneration of the index-level disc [12]. In the light of Kirkaldy-Willis model for degenerative changes in the lumbar spine and results from the present study, we posit that severe disc degeneration of the index-level disc is one of the restabilisation mechanisms available to the L5-S1 motion segment in order to mitigate increased intervertebral motions and shear stresses due to a bilateral L5 lytic defect [28]. The results also indicate that the disc height collapse and not the degenerative changes in the stiffness properties of the disc per se, play a predominant role in the restabilisation of the L5-S1 segment. With a 50% loss in disc height, the L5-S1 segment became less mobile, offloaded the ILL fibres, and experienced lower shear stresses compared with the intact disc height models. No significant difference in results was noted between M-DEG-COL LYTIC and S-DEG-COL LYTIC models. The collapsed disc height models, however, experienced high normal stresses compared with the intact disc height models suggesting that loss in disc height redistributed loads within the motion segment, decreasing the shear component of the intradiscal forces and increasing the normal component.

Over the age of 25 years, the prevalence of index-level disc degeneration in lytic defect patients was found to be significantly greater compared with an age-and-sex matched control group [11]. Although progression of a lytic defect to spondylolisthesis in adults depends on numerous factors such as spinopelvic balance, lower-lumbar muscle strength, repetitive flexion-extension activities, strength of the iliolumbar ligament; the most important structure resisting the vertebral slippage is the index-level disc [4, 6, 29, 30]. The health (or lack thereof) of the index-level disc is therefore critical to resisting the vertebral slippage. Two distinct pathways may be available to a L5-S1 segment with a bilateral lytic defect and a mildly degenerate index-level disc:

1. Within the limits of biomechanical stability provided by the active and passive spinal elements, the index-level disc may progressively lose height resulting in a decrease in intervertebral motions and redirection of the intradiscal shear stresses to normal stresses, until biomechanical stabilisation is achieved.
2. Beyond the limits of biomechanical stability provided by the active and passive spinal elements, the defect may progress to spondylolisthesis followed by a loss in disc height, in small and closed-loop increments, until biomechanical stabilisation is achieved.

From the onset of a bilateral L5 lytic defect, the exact duration of unstable and restabilisation phases may vary from patient to patient. Why some L5-S1 motion segments will adopt one pathway over the other may depend on genetic, anatomical, and lifestyle factors, and remains to be clinically explored. If the slippage progression is relatively rapid and intractable radicular pain associated with nerve root entrapment is evident, surgical intervention may be necessary to achieve biomechanical stability [12].

Progressive degeneration of the intervertebral disc is characterised by a cascade of cellular, biochemical, morphological, and functional changes. The sequence of these changes and the interplay between them are largely unknown. In the present study, a rather simplified approach was used to model progressive degeneration of the L5-S1 disc, altering only the structure (height) and material properties. The material properties of the AF and NP tissues were altered for different grades of disc degeneration in accordance with published data [25]. Whilst the shear modulus for the AF tissues was increased with progressive degeneration, Poisson’s ratio for the NP tissues was decreased to simulate the loss in incompressibility with progressive degeneration. [25] A 50% loss in mid-sagittal plane disc height was modelled to simulate a collapsed disc.

Some limitations to this study were noted. Poroelasticity and degeneration related structural defects (Schmorl’s node, annular tears, nuclear clefts, and rim lesions) in the disc were not modelled, which may have a bearing on the results. Instead of assembling multiple FE models representing a gradual change in the disc height, only a 50% collapse in disc height was modelled. The AF was modelled as a composite structure with only four concentric layers of collagen fibres (without translamellar bridges) embedded within a homogenous ground substance.

## Conclusions

In the presence of a bilateral L5 spondylolytic defect, a mildly degenerate index-level disc experienced greater intervertebral motions and shear stresses compared with a severely degenerate index-level disc during flexion and extension bending. Disc height collapse, with or without severe degenerative changes in the stiffness properties of the disc, is one of the plausible restabilisation mechanism available to the L5-S1 motion segment to mitigate increased intervertebral motions and shear stresses due to a bilateral L5 lytic defect.

## Additional file

Additional file 1: Mild (not severe) disc degeneration is implicated in the progression of L5 spondylolysis to spondylolisthesis. This supplementary information file contains data on material property values assigned to various elements used in building the five FE models of lumbosacral spine in this study. (DOCX 36 kb)

## Abbreviations

AF: Annulus Fibrosus
ALL: Anterior Longitudinal Ligament
CL: Capsular Ligament
CT: Computed Tomography
DICOM: Digital Imaging and Communications in Medicine
DOF: Degrees of Freedom
Ex: Extension
FE: Finite Element
Fx: Flexion
GPa: Giga Pascals
ILL: Iliolumbar Ligament
INTACT: An intact finite element model of L1-S1 spine without any defect or degeneration
IPT: Interpedicular Travel
ISL: Interspinous Ligament
ITL: Intertransverse Ligament
L1: First Lumbar Vertebra
L5: Fifth Lumbar Vertebra
LF: Ligamentum Flavum
LSL: Lumbosacral Ligament
Lytic: A Spondylolytic Defect
M-DEG LYTIC: A finite element model of L1-S1 spine with a bilateral L5 lytic defect and mild degenerative changes in the stiffness properties of the L5-S1 disc
M-DEG-COL LYTIC: A finite element model of L1-S1 spine with a bilateral L5 lytic defect, mild degenerative changes in the stiffness properties of the L5-S1 disc and 50% disc height collapse
mm: Millimetre
Nm: Newton-Metre
NOR LYTIC: A finite element model of L1-S1 spine with a bilateral L5 lytic defect and a normal L5-S1 disc
NP: Nucleus Pulposus
PLL: Posterior Longitudinal Ligament
RoM: Range of Motion
S-COL LYTIC: A finite element model of L1-S1 spine with a bilateral L5 lytic defect, severe degenerative changes in the stiffness properties of the L5-S1 disc and 50% disc height collapse
SSL: Supraspinous Ligament
UNSW: University of New South Wales
