# Supplementary material for: Mild (not severe) disc degeneration is implicated in the progression of bilateral L5 spondylolysis to spondylolisthesis
Source: BMC Musculoskelet Disord. 2018 Apr 2;19:98. doi: 10.1186/s12891-018-2011-0 (PMC5879802; doi:10.1186/s12891-018-2011-0)
Supplement: Supplementary file 1 — Mild (not severe) disc degeneration is implicated in the progression of L5 spondylolysis to spondylolisthesis. This supplementary information file contains data on material property values assigned to various elements used in building the five FE models of lumbosacral spine in this study. (DOCX 36 kb) [file 12891_2018_2011_MOESM1_ESM.docx]

**Mild (not severe) disc degeneration is implicated in the progression of L5 spondylolysis to spondylolisthesis**

**Table S1**. Uncalibrated material property values assigned to various elements in the finite element models. Due to lack of published data on the Iliolumbar and the Lumbosacral ligaments, these were assigned the same material properties as the Anterior Longitudinal ligament.

|  | **Element type** | **Material model** | **Material property values (uncalibrated)**  E : Elastic Modulus (MPa)  G: Shear Modulus (MPa)  ν: Poisson’s Ratio  d: Diameter (millimetres)  k: Stiffness (N/mm)  ε: Strain % | **Source literature** |
| --- | --- | --- | --- | --- |
| **Cancellous bone** | 4-noded tetrahedral | Orthotropic | E_xx = 140;  E_yy = 140;  E_zz = 200;  G_xy = 48.3;  G_yz = 48.3;  G_xz = 48.3;  ν_xx = 0.45;  ν_yy = 0.315;  ν_zz = 0.315 | [[1](#_ENREF_1)] |
| **Cortical bone** | 4-noded tetrahedral | Orthotropic | E_xx = 11300;  E_yy = 11300;  E_zz = 22000;  G_xy = 3800;  G_yz = 5400;  G_xz = 5400;  ν_xx = 0.484;  ν_yy = 0.203;  ν_zz = 0.203 | [[1](#_ENREF_1)] |
| **Endplates inner** | 4-noded tetrahedral | Isotropic | E = 2000 | [[2](#_ENREF_2)] |
| **Endplates middle** | 4-noded tetrahedral | Isotropic | E = 6000 | [[2](#_ENREF_2)] |
| **Endplates outer** | 4-noded tetrahedral | Isotropic | E = 12000 | [[2](#_ENREF_2)] |
| **Intertransverse ligaments** | Beam | Non-linear elastic | N = 16 per level  d = 1.0  E_t_ = 10.0(ε< 18%), 58.7(ε>18%);  ν = 0.3 | [[3](#_ENREF_3)] |
| **Iliolumbar ligaments** | Beam | Non-linear elastic | N = 20  d = 1.0  E_t_ = 7.8(ε< 12%), 20.0(ε>12%);  ν = 0.3 | No literature available |
| **Lumbosacral ligaments** | Beam | Non-linear elastic | N = 22  d = 2.0  E_t_ = 7.8(ε< 12%), 20.0(ε>12%);  ν = 0.3 | No literature available |
| **Zero-Gap elements** | Beam | Non-linear Zero Gap | k = 5000 | [[4](#_ENREF_4)] |

**Table S2**. Calibrated material property values assigned to various elements in the finite element models.[[5](#_ENREF_5)]

|  | **Element type** | **Material model** | **Material property values (calibrated)**  E: Elastic Modulus (MPa)  K: Bulk Modulus (MPa)  ν: Poisson’s Ratio  d: Diameter (millimetres)  k: Stiffness (N/mm)  ε: Strain %  C_1_, C_2_: Mooney-Rivlin constants  T: Tension (N) |
| --- | --- | --- | --- |
| **Nucleus pulposus** | 4-noded tetrahedral | Mooney-Rivlin, 2 parameters | C_1_ = 0.006;  C_2_ = 0.0045;  K = 105 |
| **Annulus ground substance**   1. **Anterior** 2. **Posterior** 3. **Lateral** 4. **Anterolateral** 5. **Posterolateral** | 4-noded tetrahedral | Mooney-Rivlin, 2 parameters | 1. C_1_ = 0.0672, C_2_ = 0.0168, K = 1.68 2. C_1_ = 0.0476, C_2_ = 0.0119, K = 1.19 3. C_1_ = 0.0364, C_2_ = 0.0091, K = 0.91 4. C_1_ = 0.0476, C_2_ = 0.0119, K = 1.19 5. C_1_ = 0.0459, C_2_ = 0.0115, K = 1.15 |
| **Annulus Fibres**   1. **Layer 1** 2. **Layer 2** 3. **Layer 3** 4. **Layer 4** | Beam | Non-linear elastic | 1. E_t_ = 275, ν = 0.3 2. E_t_ = 242.5, ν = 0.3 3. E_t_ = 210, ν = 0.3 4. E_t_ = 180, ν = 0.3 |
| **Ligaments**   1. **ALL** 2. **PLL** 3. **LF** 4. **CL** 5. **ISL** 6. **SSL** | Beam | Non-linear elastic | 1. N = 14 continuous, d = 4.8,   E_t_ = 23.4(ε< 12%), 60(ε>12%), ν = 0.3   1. N = 6 continuous, d = 0.7,   E_t_ = 5(ε< 11%), 10(ε>11%), ν = 0.3   1. N = 18 per level, d = 1.1,   E_t_ = 15(ε<6.2%), 10(ε>6.2%), ν = 0.3   1. N = 48 per level, d = 0.8,   E_t_ = 7.5(ε< 25%), 32.9(ε>25%), ν = 0.3   1. N = 9 per level, d = 1.2,   E_t_ = 10(ε< 14%), 11.6(ε>14%), ν = 0.3   1. N = 4 continuous, d = 1.5,   E_t_ = 8(ε< 20%), 15(ε>20%), ν = 0.3 |
| **Point-Contact elements** | Beam | Non-linear Tension Contact | k = 25  T = 5 |

ALL: Anterior Longitudinal Ligament; PLL: Posterior Longitudinal Ligament; LF: Ligamentum Flavum; CL: Capsular Ligament; ISL: Interspinous Ligament; SSL: Supraspinous Ligament

|  | **INTACT** | | **NOR LYTIC** | | **M-DEG LYTIC** | | **M-DEG-COL LYTIC** | | **S-COL LYTIC** | |
| --- | --- | --- | --- | --- | --- | --- | --- | --- | --- | --- |
|  | **C_1_ (MPa)** | **C_2_ (MPa)** | **C_1_ (MPa)** | **C_2_ (MPa)** | **C_1_ (MPa)** | **C_2_ (MPa)** | **C_1_ (MPa)** | **C_2_ (MPa)** | **C_1_ (MPa)** | **C_2_ (MPa)** |
| **Annulus Anterior** | 0.0672 | 0.0168 | 0.0672 | 0.0168 | 0.084 | 0.021 | 0.084 | 0.021 | 0.1008 | 0.0252 |
| **Annulus Posterior** | 0.0476 | 0.0119 | 0.0476 | 0.0119 | 0.0595 | 0.014875 | 0.0595 | 0.014875 | 0.0714 | 0.01785 |
| **Annulus Lateral** | 0.0364 | 0.0091 | 0.0364 | 0.0091 | 0.0455 | 0.011375 | 0.0455 | 0.011375 | 0.0546 | 0.01365 |
| **Annulus Anterolateral** | 0.0476 | 0.0119 | 0.0476 | 0.0119 | 0.0595 | 0.014875 | 0.0595 | 0.014875 | 0.0714 | 0.01785 |
| **Annulus Posterolateral** | 0.04592 | 0.01148 | 0.04592 | 0.01148 | 0.0574 | 0.01435 | 0.0574 | 0.01435 | 0.06888 | 0.01722 |

Mooney-Rivlin material model was used to simulate mechanical response of the annulus fibrosus (ground substance) and the nucleus pulposus.[[6](#_ENREF_6)] Table S3 outlines material property values assigned to the brick elements representing the annulus fibrosus in different FE models to simulate progressive degeneration.

**Table S3.** Regional variation in stiffness properties of the annulus fibrosus was modelled by dividing the annulus into five regions (anterior, anterolateral, lateral, posterolateral, and posterior). Alterations were made to the Mooney-Rivlin material model coefficients (C_1_ and C_2_) for the brick elements representing annulus fibrosus, in order to simulate mild and severe degeneration of the L5-S1 disc.

|  | **INTACT** | **NOR LYTIC** | **M-DEG LYTIC** | **M-DEG-COL LYTIC** | **S-COL LYTIC** |
| --- | --- | --- | --- | --- | --- |
| ***v*** | 0.4999 | 0.4999 | 0.4336 | 0.4336 | 0.3316 |
| **K (MPa)** | 105 | 105 | 0.1581 | 0.1581 | 0.0623 |

Table S4 outlines material property values assigned to the brick elements representing the nucleus pulposus in different FE models to simulate progressive degeneration.

**Table S4.** Alterations were made to the Poisson’s Ratio (*v*) of the brick elements representing the nucleus pulposus (causing subsequent changes to the Bulk Modulus (K)), in order to simulate mild and severe degeneration of the L5-S1 disc.

## References

1. Lu YM, Hutton WC, Gharpuray VM. Do bending, twisting, and diurnal fluid changes in the disc affect the propensity to prolapse? A viscoelastic finite element model. Spine. 1996;21(22):2570-9.

2. Polikeit A, Ferguson SJ, Nolte LP, Orr TE. The importance of the endplate for interbody cages in the lumbar spine. European Spine Journal. 2003;12(6):556-61.

3. Kiapour A, Ambati D, Hoy RW, Goel VK. Effect of graded facetectomy on biomechanics of Dynesys dynamic stabilization system. Spine. 2012;37(10):E581-9.

4. Strand7. Theoretical background to the Strand7 finite element analysis system. Strand7 Pty Ltd; 2005. p. Strand7 Release 2.3.

5. Ramakrishna VAS, Chamoli U, Viglione LL, Tsafnat N, Diwan AD. The Role of Sacral Slope in the Progression of a Bilateral Spondylolytic Defect at L5 to Spondylolisthesis: A Biomechanical Investigation Using Finite Element Analysis. Global Spine Journal. 2017;In Press.

6. Boyce MC, Arruda EM. Constitutive Models of Rubber Elasticity: A Review. Rubber Chemistry and Technology. 2000;73(3):504-23.
